# Bioethics and xenotransplantation from pig to human

**DOI:** 10.1016/j.clinsp.2023.100170

**Published:** 2023-02-03

**Authors:** Flavio Henrique Ferreira Galvao, Max Grinberg

**Affiliations:** a Departamento de Gastroenterologia, Disciplina de Transplanre de Órgãos do Aparelho Digestivo, Laboratório de Investigação Medical (LIM) 37, Hospital das Clínicas, Faculdade de Medicina, Universidade de São Paulo (USP), São Paulo, SP, Brazil; b Departamento de Cardiologia, Instituto do Coração Hospital das Clínicas, Faculdade de Medicina, Universidade de São Paulo (USP), São Paulo, SP, Brazil; c Bioethical Committee from Hospital das Clínicas, Faculdade de Medicina, Universidade de Sao Paulo (USP), São Paulo, SP, Brazil

Xenotransplantation is defined as the transplantation of organs, tissues, and cells between organisms of different species. The use of organs from these animals would theoretically reduce the important mortality on transplant lists worldwide due to the lack of donors. Furthermore, the availability of using healthy animals as donors would improve transplant outcomes because it would avoid the deleterious effects of brain death and prolonged preservation of organs present in the deceased human donor.^1^, ^2^, ^3^ Xenotransplantation would also be interesting to treat diseases for which human organ allotransplants are not traditional therapies (e.g., insulin-dependent diabetes mellitus, chronic pain syndromes epilepsy, and degenerative diseases such as Parkinson's and Huntington's disease).^1^, ^2^, ^3^, ^4^, ^5^

Swine is a species with great potential to be an organ donor because they have organs morphologically and physiologically compatible with those of humans; nevertheless, this method may cause hyperacute rejection, an immunological reaction caused by pre-formed antibodies that destroy the transplanted organs in a few hours and are always present in transplantation between species very different (discordant xenotransplantation).^1^, ^2^, ^3^ This catastrophic immunological reaction was responsible for the failures of the first xenotransplants performed using organs and tissues from non-genetically modified animals in the 60s and 70s and caused the abandonment of this methodology.^2^^,^^4^ Hyperacute rejection is always a very serious complication without effective treatment that requires immediate graft removal to save the recipient.^5^, ^6^, ^7^

The evolution of genetic modulation of living beings using technologies such as CRISPR/Cas9 (Clustered Regularly Interspaced Short Palindromic Repeats) provide the creation of genetically modified pigs whose organs would be immunologically compatible with human, escaping from hyperacute rejection.^8^ Research using an organ from these modified animals transplanted in non-human primates accomplished promising results, especially in terms of survival and function.^9^

These progresses revived the interest in clinical xenotransplantation and brought great hope to thousands of patients on the transplant list and inspired optimistic expectations in several researchers. Shekhar AC, from the Center for Bioethics at Harvard University (USA), believes that the use of organs from these animals would solve the bioethical challenges associated with post-brain-death donation and the risks of living donation.^10^ Prof. Silvano Raia, Professor Emeritus at HC-FMUSP and a world pioneer in liver transplantation recently leads a research group that aim to build in Brasil a vivarium specializing in the production of genetically modified pigs to serve as organ transplant donors.^11^ The group of digestive organs transplants at HC-FMUSP suggested in experimental research that xenotransplantation using these animals would be the ideal solution for patients listed for multivisceral transplantation (a modality where the stomach, intestine, pancreas, and liver are transplanted simultaneously), as these candidates have a long waiting time on the list because they compete with patients from various transplant lists (liver, pancreas, and intestine).^6^^,^^7^^,^^12^^,^^13^

Recently, three cases of xenotransplantation in humans using genetically modified swine were reported in the United States of America. Two of them were kidneys xenograft from genetically modified pigs transplanted into brain-death patients. The recipients' circulatory and respiratory activity was maintained on ventilators for a period of 54 hours and then submitted to euthanasia. In both cases, creatinine levels decreased to normal levels after reperfusion with normal urine production during the period of the experiment, showing a good function of the transplanted kidneys, without the presence of rejection. Thus, the kidney xenograft escapes from the normal period of xenograft destruction by hyperacute rejection.^14^

The other was heart xenotransplantation from genetically modified swine in a critical recipient without indication for usual heart allotransplantation (human-to-human transplantation). This was the first xenotransplantation in live recipients, using a genetically modulated swine organ and the bioethics approval commission for this xenotransplantation was based on compassion. The recipient survived for 60 days and had a Cytomegalovirus (CMV) infection from the donor swine as the main complication.^15^ Alternatively, a problematic reaction to an intravenous human Immunoglobulin (IVIg) (antibody-mediated rejection drug), or humoral rejection might have contributed to the patient's heart failure. Yet, the precise basis of the recipient's death continues to be uncertain and remains under evaluation.^16^ The porcine donor in this case had 10 genetic modulations (knock out of three porcine genes that promote hyperacute rejection, knock out of one gene that causes continued organ growth, and six human genes that promote tolerance (rejection reduction) were inserted (knock-in) in the porcine genome.^17^

However, despite these advances and optimism, bioethical issues related to xenotransplantation remain polemic and some issues deserve particular attention. Clinical application of innovation such as xenotransplantation raises questions concerning the balance between beneficence, individual autonomy and the recognition of possible damage to society and the environment.^18^^,^^19^ The problems of autonomy in xenotransplantation are related to the difficulty of composing the free and informed consent form, since the risks and benefits for the recipient, given the complexity of the situation, are still unknown. Furthermore, some of the greatest risks of this procedure, zoonosis, is associated with the possibility of its success. Therefore, the recipient's notion of being able to withdraw from participating in the research at any time must be very well clarified before proceeding with the xenotransplantation, if there is a predictable chance of survival for a long period.^18^^,^^19^

The risk of xenotransplant recipients contracting and transmitting Porcine Endogenous Retroviruses (PERV) infections is another bioethical issue that represents a potential public health problem because could cause a pandemic. This xenozoonosis or xenosis would be hazardous not only to the recipient but also to their contacts who would not even be aware of this situation.^20^^,^^21^ This risk of pandemic may be reduced by removing the PERV from the swine donors by CRISPR/Cas9 and other technologies; nevertheless, Innovative development of new microbiological assays and microbiological techniques should be applied to diagnose and prevent infection in xenograft recipients. In fact, swine xenotransplant recipients and their contacts probably will need lifelong surveillance to detect and treat any unexpected disease early and protect society as a whole.^20^^,^^21^

The appropriate care of the swine that will be a donor in the xenotransplantation is another important bioethical issue. The swine has less social rejection for use as a donor due to the fact that this species is normally bred and slaughtered for food practically all over the world. However, according to Dr. Entwistle from Thomas Jefferson University (Philadelphia, USA), confinement, social isolation (to avoid infections), and repeated procedures such as blood collections, which are part of the protocol for producing these animals, violate guidelines for the care and use of laboratory animals and deserves, at the very least, discussion and open public debate.^18^

The bioethical enigmas from xenotransplantation arouse philosophical questions that will be discussed below.

Since Hippocrates, the human being has assumed responsibilities with another human being to deal with health/disease issues. Currently, fiction is realized as robots, cyborgs, and chimeras. Technology (a technique provided by science) is precisely allowing the expansion of the integration of Homo sapiens to Nature. There have been centuries of this integration into the plant world, pharmaceuticals, for example. Integration with the animal is essentially food, but also transport (horses), safety (guard dog, visually impaired dog), and Health (leeches are still used in reconstructive surgeries to prevent thrombosis).

Since the use of porcine tissue bioprostheses, which was abandoned especially for infectious aspects, transhumanism is related to the treatment of several diseases. The cyborg is there, those who have a coronary stent, implantable defibrillator, or other therapeutic equipment are people analogous to cyborgs. In fact, the defibrillator is independent of the person and acts by itself. The potential of xenotransplantation is an evolutionary variation of transplantation between humans because it not only expands the potential of donor availability, but also does not depend on the donor's will, but on various laws, including those related to the protection of animals.

The pig, unlike the non-human great primates, seems to be the element of nature that best fits the human being to serve as a donor. Despite the greater immunological and physiological similarity with humans, the use of non-human primates in xenotransplantation has strong social rejection, mainly because these animals are in the process of extinction. Apart from the aspect of technoscience and morality duality, of “Should/Can/Want?”, science ventures, create paths and after (or before and during) evidently everything needs to be evaluated, in which bioethics has a strong contribution.

As current biotechnology still does not make it possible to inject stem cells and create a new organ, one idea is to implant an organ already formed from swine. admissible? Feasible? resolute? Some questions are primary: 1) Technically possible, in the sense of physiological and anatomical adaptation?; 2) Immune tolerability to prevent hyperacute rejection?; Some kind of swine could be more convenient?; 3) Evolutionary aspect, the post-modern history procedure, because in every situation of complexity, no matter how much there is a maximum rigor, it is always necessary to leave an opening for the unknown, the imponderable, the inevitable. Comorbidities exist and influence. conflicts of interest too. It is known how it starts, but not how it will develop. At the beginning of making the bovine pericardium bioprosthesis, surgeons went to the death verification services, selected perfect retail and they made the prosthesis. Then others also began to capture and do, which caused quality control concerns & quot; amateur & quot; because of course there is a learning curve, especially in artisanal. Ideological aspects are predictable to happen, how much the animals would be available to the man, just as there has already been a situation where parents chose to have a child with the aim of using their cells to help another sick son. Dilemmas, challenges, and conflicts are inevitable and there will be those who are radically in favor and radically against innovative means of treatment of various diseases. The big question is: As well as being human domesticated animals in a utilitarian attitude to serve, what are the premises of today's society for this service that obligatorily kills the animal for humans to survive a disease? Similar to slaughter for food purposes?^22^ It certainly implies contrapositions of collective vision (morality) and individual, ethical vision for those in need.

The Frenchman Claude Lévi-Strauss, one of the great thinkers of the 20^th^ century (1908‒2009), stated that human organ transplantation is a type of cannibalism in a broad concept that is not reduced to a macabre meal, but to the introduction into the body of a human being, from parts of the body of another human being. In the current context, this situation is also present in xenotransplantation.

While in man it became natural for there to be a transfer of organs through medicine, but not through gastronomy, in the case of the use of pork, for example, the opposite occurs, it is natural in gastronomy and brings an unsettling feeling if used by therapeutic medicine. for transplant. The notion of chimera, which we do not have when we eat, and our muscles, for example, acquire proteins from animals and lose their presence through metabolism, differs from xenotransplantation. The observation by humans of the food chain in nature between animals and the notion of reproduction within species with the birth of similar beings are illustrative of how customs passed from generation to generation discourage “stopping to think” and how science is always with the offending light on. Let's imagine a newborn with a transplanted pig's heart and how it gives rise to developments in the human condition: would the affective symbolism of the heart change? Would your child's heart be 100% “human”?

The authors can state that the digestive system in humans has evolved in such a way that it allows us to circumvent the genetic aspects, that is, there is no rejection of the foreign protein due to metabolic processes. The opposite occurs with the implantation of an organ from another individual. In swine organ transplantation, the immunological barrier, even with the current genetic modifications, still represents a major limitation. However, the rapid biotechnological advances currently verified give a perspective of possible control not only of rejection but also of a possible pandemic from zoonosis through technology to remove porcine genes harmful to the human species.

Bioethics in its interfaces with humanity fulfills its transdisciplinary role, which goes beyond and across disciplines. It broadens the discussions on morality in the therapy of xenotransplants, including the issue of animal rights, in a desirable platform for discussion about creating/sacrificing/using for food and creating/sacrificing/using for scientific research. It is interesting how Bioethics encourages us to read some subjects and want to ask some questions, such as thinking about the cultural situation between transplant medicine and nutrition/gastronomy. In this way, bioethics motivates thinking and approaching difficulties in different and multidisciplinary ways, taking advantage of the intelligence of many to develop knowledge.

Finally, it is important to discuss the ethical problem of the criteria for the indication and allocation of genetically modified pigs. These first cases were performed in patients outside the regular criteria for allotransplantation (transplantation between human beings), which could compromise the success of these transplants. Another relevant aspect is the cost of the production of a genetically modified swine. The current cost will certainly restrict the indication of this procedure to people with greater economic power.

The authors conclude by stating that bioethics in xenotransplantation using genetically modified pigs presents more doubts than certainties. Society as a whole, including bioethicists, politics, and the scientific community, needs to come to some understanding of the risk/benefit calculation and make decisions and protocols about clinical xenotransplantation. This understanding must be based on scientific research with an appropriate methodology that always promotes the elucidation of the truth. The World Health Organization (WHO), The Transplantation Society (TTS), and the International Xenotransplantation Association (IXA) have been performing substantial and continuous guidance and regulations, with the medical and scientific community, in order to admit effective and safe xenotransplantation clinical trials.^23^

## Conflicts of interest

The authors declare no conflicts of interest.
